# *Peganum harmala* Polysaccharide Mitigates LPS‐Induced Inflammatory Response in Macrophages by Activating Autophagy Pathway

**DOI:** 10.1002/fsn3.4501

**Published:** 2025-01-24

**Authors:** Manzeremu Rejiepu, Jun Shen, Junqing Liang, Alaili Maitikabili, Jian Yang, Ling Zhang, Na Mi

**Affiliations:** ^1^ Clinical Medical Research Institute, the First Affiliated Hospital of Xinjiang Medical University, Xinjiang Medical University Urumqi Xinjiang China; ^2^ Basic Medical College Xinjiang Medical University Urumqi Xinjiang China; ^3^ College of Pharmacy Xinjiang Medical University Urumqi China

**Keywords:** autophagy, inflammation, LPS, macrophages, mTOR, *Peganum harmala* polysaccharide

## Abstract

*Peganum harmala,* a member of the *Zygophyllaceae* family, is known for its diverse biological activities, including anti‐inflammatory properties. The mechanisms through which *P. harmala* polysaccharide (LTP) induces autophagy, however, remain largely unexplored. This study aims to elucidate the role of LTP in autophagy induction and its efficacy in mitigating inflammation within macrophages. Autophagosome formation was evaluated using GFP‐LC3 vectors, and LC3‐II levels induced by LTP were analyzed through laser scanning confocal microscopy. Western blotting assessed the expression of autophagy‐related proteins and the phosphorylation state of p70S6K in NRK cells, treated both with and without LTP, alongside autophagy inducers and inhibitors. Additionally, RAW264.7 cells were treated with 1 μg/mL lipopolysaccharides (LPS), followed by Western blotting and ELISA assays to quantify inflammatory markers. The study's outcomes demonstrate that LTP facilitates an increase in autophagic activity, as evidenced by the enhanced expression of LC3‐II and reduced levels of p62 in both NRK and RAW264.7 macrophages. This effect is mediated through the activation of the AMPK‐mTOR signaling pathway, without inhibiting the autophagosome–lysosome fusion process. In vitro experiments with RAW264.7 cells treated with 1 μg/mL LPS showed that LTP markedly decreased the levels of TNF‐α and IL‐6. Our findings indicate that LTP effectively reduces inflammation in LPS‐stimulated macrophages by promoting autophagy via an mTOR‐dependent mechanism.

## Graphical abstract

In Xinjiang, *Peganum harmala* is a traditional medicinal plant and is widely cultivated as a Medicine‐Food plant. People add *P. harmala* to their teas and consume them for a higher immunity and a stronger body. Thus, in the present study, we explored the autophagic regulatory activity of LTP treatment and investigated its enhanced autophagy and exerted its anti‐inflammatory activity.

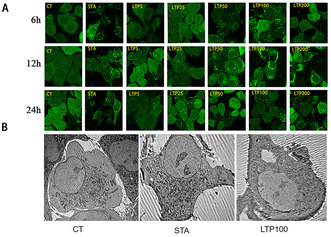

## 1 Introduction

*Peganum harmala*, with its vast distribution across the Mediterranean region of Europe, Central Asia, and South America's southern areas, is widely recognized in traditional Chinese medicine for its therapeutic potential in treating various diseases. The unique biological properties of natural products, including those derived from *P. harmala*, have recently garnered significant attention for their promising applications in medical science. Herbal products continue to provide hope for certain ailments that modern medicine is unable to cure. Numerous individuals, whether aware of their benefits or not, are utilizing these natural remedies (Keihanian et al. 2021; Zhu, Zhao, and Wang 2022). The β‐carboline alkaloids, notably harmine, derived from *P. harmala*, have showcased profound anticancer attributes. Considering the analogous mechanisms of action between the alkaloids of *P. harmala*, harmine in particular, and established anticancer pharmaceuticals, these compounds hold promise as innovative standalone treatments or as contributors to adjunctive cancer treatment regimens (Jalali, Dabaghian, and Zarshenas 2021).

Traditional chinese medicine (TCM) has demonstrated a tangible therapeutic effect in the management of Ulcerative Colitis. It offers a multifaceted approach to alleviating and treating this condition, with an emphasis on syndrome differentiation and individualized treatment as its cornerstone. By integrating the insights of modern medicine, the clinical efficacy of TCM in treating Ulcerative Colitis can be further enhanced. With the intention of augmenting research into the medicinal applications of *P. harmala*, this study aims to provide a valuable reference within the antimicrobial domain. Additionally, it seeks to establish a solid foundation for the advancement of natural antimicrobial agents as potential therapies for the treatment of infectious diseases (Liu et al. 2022).

The extracts from *P. harmala* demonstrate a wide range of biological functions, including anti‐inflammatory, antibacterial, antifungal, antiviral, antioxidant, pain‐relieving, cardioprotective, antitumor, antidiabetic, brain‐protective, misfunctioned, anti‐proliferative, and anticancer activities, among others (Fahmy et al. 2021; Jalali, Dabaghian, and Zarshenas 2021; Wang et al. 2022). Drawing upon a wealth of historical, experimental, and clinical data, *Berberis vulgaris*, *Cerasus avium*, *Berberis integerrima*, *Apium graveolens*, *P. harmala*, *Citrus aurantifolia* (lime), *Citrus aurantium* (bitter orange), *Centella asiatica*, and *Curcuma longa* have emerged as plants with significant potential in targeting therapeutic avenues for chronic heart failure. These botanicals have demonstrated the ability to enhance cardiac function, offering promise in the management of this debilitating condition (Keihanian et al. 2021).

China has a lot of native Chinese medicine. In ancient times, people began to use some Chinese herbs for pain relief, plague, and other diseases. Now for some serious diseases also, people are taking Chinese medicine to avoid surgery, such as for kidney stone.

Allium sativum or garlic has been used for many years in the traditional medical practice of various cultures to reduce the risk of several diseases, mainly of the cardiovascular system. The genus *Anchusa* (Boraginaceae family) is a traditional Uygur medicine for treating cardiovascular and cerebrovascular diseases in the Xinjiang region (Hu et al. 2020).

Autophagy, characterized as an intracellular self‐degradative phenomenon (Cao et al. 2019), has recently been recognized as a pivotal modulator of inflammatory responses. Prior investigations have elucidated its protective function in mitigating inflammation triggered by lipopolysaccharides (LPS) (Wei et al. 2020; Xiao et al. 2022). This mammalian autophagic process is integral to cytoplasmic integrity, facilitating cellular metabolism, and bolstering both innate and adaptive immune defenses (Morishita and Mizushima 2019). The term “autophagy flux” encompasses the entire autophagic continuum, beginning with phagophore initiation, autophagosome formation, subsequent fusion with lysosomes, and culminating in the degradation of the encapsulated material (Kuma, Komatsu, and Mizushima 2017). Autophagy, as a cellular catabolic pathway, offers a safeguard against exogenous challenges, including infections, and serves as a bulwark against endogenous sources of inflammation, such as impaired organelles and protein aggregates.

Recent developments have illuminated the critical function of the autophagy pathway and its constituent proteins in modulating both immunity and inflammation. These proteins are instrumental in preserving a nuanced balance between the beneficial and detrimental aspects of immunity and inflammation, thereby offering protection against a spectrum of diseases, including infectious, autoimmune, and inflammatory conditions (Deretic 2021). The modulation of autophagy has been documented as protective in scenarios of dystrophy, LPS‐induced inflammation, and oxidative stress (Kuno et al. 2018; Liu et al. 2019; Miceli et al. 2018; Pan et al. 2018). Notably, empirical evidence suggests that autophagy can increase survival rates, mitigate histological damage, reduce lung water content relative to dry weight, and decrease pro‐inflammatory cytokine levels (Wei et al. 2020; Xiao et al. 2022; Zhao et al. 2019). Furthermore, autophagy has been shown to modulate both the composition and activation of the inflammasome, leading to attenuated inflammatory responses (Peng et al. 2021). Macrophages, critical for maintaining tissue equilibrium and acting as immune surveilants, are strategically distributed throughout mammalian tissues, playing an indispensable role in health and disease (Koelwyn et al. 2018).

In the present study, we explore the protective effects of *P. harmala* polysaccharide on inflammation, which is induced by lipopolysaccharides (LPS) in macrophages, through the activation of autophagy. Our research has delineated the molecular pathway through which *P. harmala* polysaccharide augments autophagy, thereby exerting its anti‐inflammatory properties. Specifically, our observations highlight its capability to modulate autophagy in an mTOR‐dependent manner. This insight into the mechanistic pathway offers a promising avenue for therapeutic intervention in conditions characterized by excessive inflammatory responses.

## 2 Materials and Methods

### 2.1 Reagents and Antibodies

*Peganum harmala* polysaccharide (≥ 98%, HPLC) was bought from Shanghai YuanYe Bio‐Technology Co. Ltd. (Shanghai, China) and dissolved in DMSO. Dulbecco's modified eagle's medium (DMEM) was purchased from Hyclone (Logan, UT, USA). Dulbecco's phosphate‐buffered saline (DPBS) was bought from Gibco (Staley Rd. Grand Island, NY, USA), and Rapamycin, bafilomycin‐A1, and 3‐Methydenine (3‐MA) were purchased from Sigma‐Aldrich (United States). Primary antibodies include anti‐LC3 (PM036, MBL), anti‐Phospho‐p70S6K (9206, CST), and anti‐β‐actin antibody (A2066; Sigma). Secondary goat anti‐rabbit antibodies were purchased from Invitrogen (United States). ECL chemiluminescence reagents were the product of BOSTER (Wuhan, China).

### 2.2 Cell Lines and Cell Culture

The GFP‐LC3 stably expressing NRK cell line was acquired from Professor Li Yu's laboratory within the School of Life Sciences at Tsinghua University, Beijing, China. Additionally, mouse peritoneal macrophage RAW264.7 cells were sourced from the Cell Bank of the Shanghai Chinese Academy of Sciences. The GFP‐LC3 NRK cells were meticulously maintained in a controlled environment at 37°C with 5% CO_2_ and saturated humidity within an incubator. The culture medium utilized was high‐glucose DMEM, enriched with 10% fetal bovine serum and supplemented with 100 μg/mL of penicillin and streptomycin to prevent bacterial contamination. To ensure optimal growth conditions and nutrient availability, the medium was refreshed daily.

### 2.3 Quantification of Cells With GFP‐LC3 Puncta

For the imaging of GFP‐LC3 puncta, NRK cells that stably express GFP‐LC3 proteins were cultured on Lab‐Tek Chambered Cover Glasses and maintained in an environment with 5% CO_2_ at 37°C. The cells were subjected to treatment with varying concentrations of *P. harmala* polysaccharide (5, 25, 50, 100, and 200 μg/mL) for durations of 6, 12, and 24 h, respectively. Subsequently, images were captured utilizing a confocal laser‐scanning microscope to facilitate the quantification of cells displaying GFP‐LC3 puncta.

### 2.4 Immunofluorescence and Transmission Electron Microscopy Analysis

The cells underwent fixation with 4% paraformaldehyde and were subsequently blocked using 2% bovine serum albumin (BSA) for a duration of 30 min. This was followed by an overnight incubation at 4°C with the designated primary antibody. Thereafter, the cells were treated with the appropriate secondary antibody for 1 h at room temperature. Immunostained cells were visualized under a confocal microscope, employing the Alexa 488 and Alexa 546 filter sets for detection. Furthermore, to prepare for transmission electron microscopy analysis, the cells were additionally fixed in 2.5% glutaraldehyde for 1 h. Post‐fixation, the cells were processed into sections to facilitate detailed examination through transmission electron microscopy.

### 2.5 Western Blotting

Cells were lysed using a 2% sodium dodecyl sulfate (SDS) solution. Subsequently, the lysates were combined with loading buffer and subjected to heating at 100°C for 10 min to denature the proteins. The proteins were separated using SDS‐PAGE and transferred onto NC membranes (Millipore). Following transfer, the membranes were blocked for 1 h using 5% nonfat milk to prevent nonspecific binding. After blocking, the membranes were incubated with primary antibodies specific to the target proteins. ImageJ software was utilized to quantitate the integrated optical density of each protein band, following visualization of protein bands using ECL chemiluminescent reagent with the aid of HRP‐conjugated secondary antibody.

### 2.6 Macrophages RAW264.7 Inflammatory Cell Model

The RAW264.7 macrophages were seeded in a 96‐well cell culture plate at a density of 2 × 10^4^ cells per well. To induce inflammation, the LPS‐treated group was exposed to 1 μg/mL lipopolysaccharide (LPS) for 2 h. For the treatment phase, the cells were incubated in various media compositions containing different concentrations of lipoteichoic acid (LTP) specifically, 25, 50, 100, and 200 μg/mL over a period of 12 h.

### 2.7 Detection of Autophagy and Inflammation in the Macrophagy RAW264.7

RAW264.7 cells were seeded in 6‐well plates and incubated overnight at 37°C in a humidified atmosphere containing 5% CO_2_. The experimental setup included a control group, which received 2000 μL of basic medium (Control CT), and various treatment groups as follows: (1) A model group treated with 2000 μL of basic medium supplemented with 1 μg/mL lipopolysaccharide (LPS) to induce inflammation. (2) A group treated with 100 μg/mL of LTP in a basic medium to assess its effect on the cells independently. (3) A group receiving basic medium supplemented with both 1 μg/mL LPS and 100 μg/mL LTP, to evaluate the combined effects of LPS‐induced inflammation and LTP treatment. (4) A group treated with 100 μg/mL LTP and 50 μg/mL Rapamycin in basic medium, to investigate the synergistic effects on autophagy induction. (5) A group receiving 100 μg/mL LTP and bafilomycin A1 in basic medium, aimed at studying the interaction between LTP treatment and autophagy inhibition. (6) Finally, a group treated with 100 μg/mL LTP and 3‐methyladenine in basic medium, to further explore autophagic flux modulation. Following the addition of the respective drug‐supplemented media to each well, the cells were cultured for 12 h. Subsequently, protein extraction was performed for downstream analyses to determine the levels of autophagy and inflammation.

### 2.8 Statistical Analysis

All data were analyzed using SPSS Software. All samples were carried out in triplicate, and triplicate data were expressed as means ± SD, for the three different experiments. One‐way ANOVA was used to measure statistical differences between the means within each experiment. The significant statistical difference of *p* value *p* < 0.05 was used.

## 3 Results

### 3.1 LTP Induces Autophagy In Vitro

**FIGURE 1:**
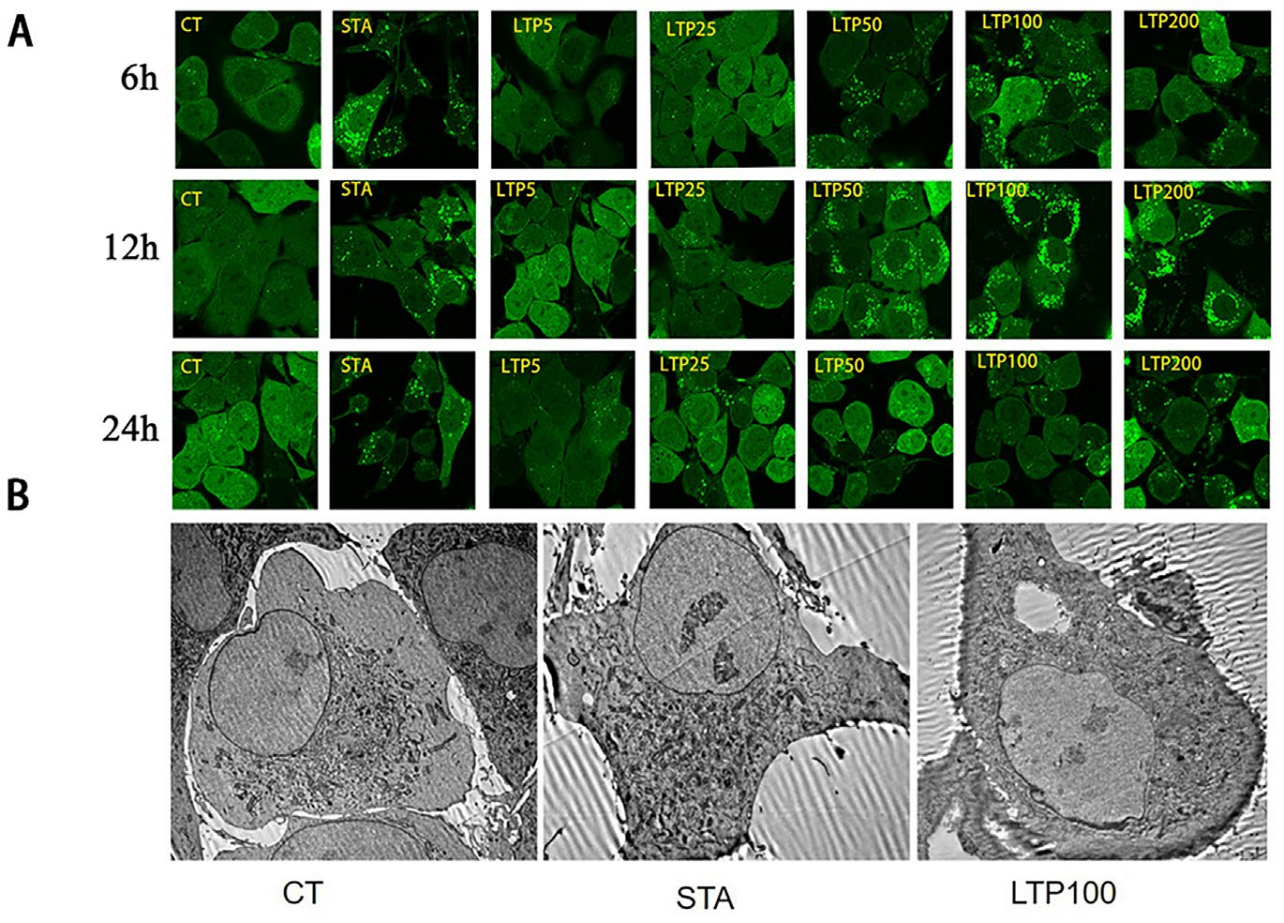
LTP increases the formation of autophagosomes. (A) The NRK cells stably expressing GFP‐LC3 were treated with or without varying concentrations (25, 50, 100, 200 μg/mL) of LTP for 6, 12, 24 h and cells were observed using confocal microscope. (B) Autophagosomes in NRK cells under electron microscope.

In this study, we quantified the accumulation of GFP‐LC3 puncta in NRK cells utilizing confocal microscopy to assess autophagy induction. Cells devoid of any intervention served as the blank control group (CT), while the starvation model was established by treating cells with Dulbecco's Phosphate Buffered Saline (DPBS) for a duration of 2 h, to simulate nutrient deprivation. Experimental groups were subjected to varying concentrations of LTP (5, 25, 50, 100, and 200 μg/mL) for periods of 6, 12, and 24 h, respectively (Figure 1A). In starved cells, there was a remarkable increase observed in the number of GFP‐LC3 puncta when compared to control cells. Additionally, the LTP 100 μg/mL group exhibited a significant augmentation in the number of GFP‐LC3 puncta, demonstrating a notable disparity from the other groups. Moreover, the number of GFP‐LC3 puncta in LTP100 μg/mL 12 h group was significantly increased compared with the control group. These results collectively suggest that the administration of 100 μg/mL of LTP effectively induces autophagy, thereby warranting its selection for subsequent treatment in this study.

### 3.2 LTP Induces Autophagy Through a mTOR‐Dependent Manner

**FIGURE 2:**
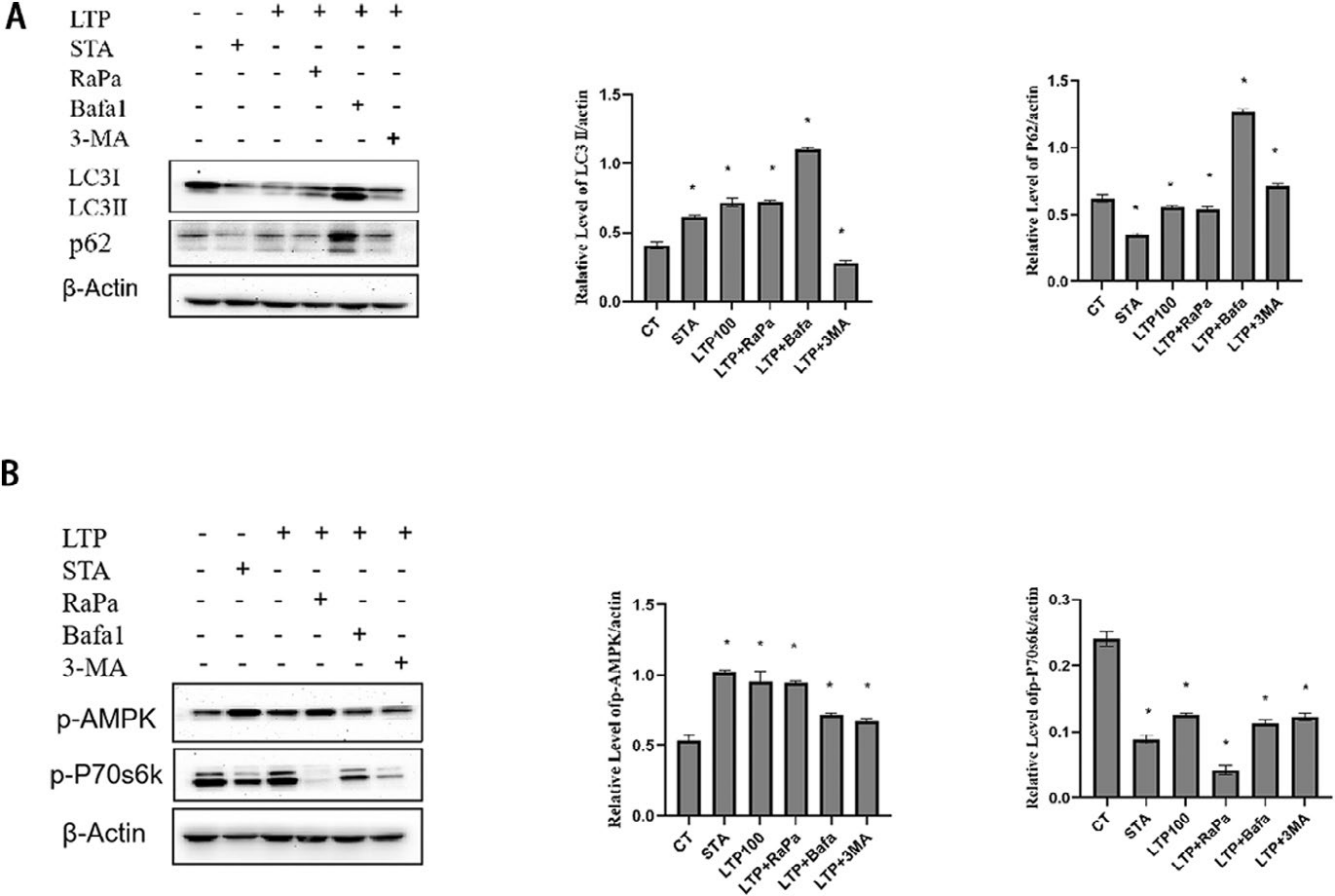
LTP induces autophagy in mTOR‐dependent manner. (A) The NRK cells were treated with or without 100 μg/mL LTP, 100 nM rapamycin, 50 nM BafilomycinA1, 5 mM 3 MA, or both for 12 h and the cell extracts were incubated with antibodies against LC3 and p62 proteins. (B) Statistical analysis of p‐p70s6k and p‐AMPK protein expression in NRK cells after treated with or without 100 μg/mL LTP, 100 nM rapamycin, 50 nM BafilomycinA1, 5 mM 3 MA, or both for 12 h.

To substantiate our preliminary findings, we employed Western blot analysis to ascertain the expression levels of the autophagy markers LC3 and p62. The results indicated a notable increase in the LC3‐II level, while a decrease in the p62 level was observed in LTP‐treated group. LTP combined with Bafilomycina1(BafA1), an autophagosome–lysosome fusion inhibitor, resulted in the prevention of fusion, leading to a significant accumulation of LC3‐II. Upon treatment of cells with LTP and 3‐methyladenine (3‐MA), the expression level of LC3‐II was markedly reduced. In order to investigate whether LTP‐induced autophagy is mTOR dependent or not, we treated NRK cells with or without LTP, Rapamycin, BafilomycinA1 and 3 MA, or both, and performed immunoblotting analysis for detection of endogenous LC3‐II, predicting that LTP and Rapamycin would have additive effects in inducing autophagy on mTOR dependent way. We further tested the phosphorylation levels of p70S6K, a substrate of mTOR, inhibited during induction of autophagy. Thus, we detected the phosphorylation levels of p70S6K by Western Blotting analysis. LTP reduced phosphorylation levels of p70S6K, further indicating that LTP might inhibit the mTOR pathway (Figure 2).

### 3.3 LTP Does Not Impede Autophagosome–Lysosome Fusion

Autophagy is the main intracellular degradation system, inhibition of the autophagic degradation pathway can lead to the accumulation of autophagosomes within cells. To investigate the impact of LTP on the fusion between autophagosomes and lysosomes, we performed an immunofluorescence assay that allowed us to simultaneously observe LC3 and LAMP1. Following exposure to LTP, there was a notable rise in the quantity of autophagosomes (indicated by the color red) and a significant co‐localization with lysosomes (indicated by the color green) when compared to the control cells. Treatment with LTP resulted in an increase in autophagic structures within the LTP‐treated cells, with the majority of these structures identified as autolysosomes. These findings provide evidence that the augmented autophagosome count induced by LTP is attributed to the induction of autophagy rather than the prevention of autophagosome degradation.

### 3.4 LTP Could Induce Autophagy in Macrophages

Following LTP treatment, we examined the levels of LC3 and p62 expression in RAW264.7 macrophage cell lines. In LTP‐treated cells, there was a significant increase in the level of LC3‐II, and it was evident that p62 degradation occurred. Furthermore, the accumulation of LC3‐II was observed when LTP was combined with Bafilomycin A1 (BafA1), an autophagosome–lysosome fusion inhibitor. Conversely, the expression level of LC3‐II decreased significantly when cells were treated with LTP and 3‐methyladenine (3‐MA). From the data obtained, it was revealed that LTP has the ability to induce autophagy in macrophages.

### 3.5 LTP Suppresses the Inflammatory Response by Inducing Autophagy in Macrophages

Autophagy has been previously established as a pivotal mechanism for attenuating inflammation within macrophages, serving as a critical process for the degradation and recycling of cellular components. In this context, we aimed to delineate the potential anti‐inflammatory effects of (LTP) on macrophage‐mediated immune responses. Our experimental outcomes revealed a significant diminution in the expression levels of pro‐inflammatory cytokines, specifically tumor necrosis factor‐alpha (TNF‐α) and interleukin‐6 (IL‐6), within the cohort receiving LTP treatment. This contrasted with the observed upregulation of TNF‐α and IL‐6 expression post‐treatment with 3‐methyladenine (3‐MA), an autophagy inhibitor, while treatment with rapamycin, an autophagy inducer, led to a reduction in these cytokines. These findings collectively underscore the capacity of LTP to mitigate inflammatory responses in macrophages, primarily through the induction of autophagy. This mechanistic insight into LTP anti‐inflammatory action provides a novel perspective on its therapeutic potential in modulating immune responses.

## 4 Discussion

Natural products often display unique biological properties. *P. harmala*, a medicinal plant, has demonstrated clinical efficacy in the treatment of ailments such as cough, hypertension, diabetes, jaundice, colic, malaria, Alzheimer's disease, and other human conditions (Zhu, Zhao, and Wang 2022). There has been a gradual rise in research documenting the antibacterial, antifungal, antiviral, and antiparasitic effects of *P. harmala*. Numerous studies have revealed that the constituents derived from *P. harmala* and its derivatives have the ability to hinder the growth of various microorganisms. They achieve this by stimulating the accumulation of reactive oxygen species (ROS) within the microorganisms, impairing cell membranes, augmenting cell wall thickness, disrupting cytoplasmic structures, and interfering with DNA synthesis (Li, Cheng, and Wang 2017; Zhu, Zhao, and Wang 2022).

Macrophages are integral to the immune system, serving not only in the removal of waste materials but also playing a pivotal role in tissue regeneration. These cells are typically categorized into two primary types: classically activated (M1) macrophages, which are proinflammatory, and alternatively activated (M2) macrophages, which exhibit anti‐inflammatory properties. The distinction between M1 and M2 macrophages is derived from their surface markers, functional roles, and the specific factors they produce. This classification underscores the complexity and versatility of macrophages in immune responses and highlights their potential as targets for therapeutic interventions in a wide array of diseases characterized by inflammation and tissue damage. M1 macrophages secrete pro‐inflammatory cytokines, including but not limited to TNF‐α, IL‐1α, IL‐1β, IL‐6, IL‐12, IL‐23, and cyclooxygenase‐2 (COX‐2), while displaying a lower production of IL‐10. These cytokines collectively contribute to the inflammatory response. In contrast, the generation of M2 macrophages is facilitated by the influence of type 2 T‐helper cell (Th2) cytokines, specifically IL‐4 and IL‐13. M2 macrophages demonstrate a decreased production of IL‐12 but exhibit elevated levels of IL‐10 and TGF‐β cytokines, which promote anti‐inflammatory effects (Kim et al. 2021; Viola et al. 2019).

Lipopolysaccharide (LPS), a critical constituent of the outer membrane of gram‐negative bacteria, triggers a cascade of inflammatory responses in pulmonary epithelial cells, as evidenced by recent research (Li et al. 2019). In contrast to the autophagy inhibitors, the autophagy activity was hindered and LPS‐induced acute lung injury was reversed with the administration of 3‐methyladenine (Ding et al. 2018). This occurred as a result of inhibiting both inflammation and autophagy. Macrophages, key players in the inflammatory process, are centrally involved in initiating inflammatory responses. Lipopolysaccharide (LPS), a component of the cell wall of Gram‐negative bacteria, is instrumental in triggering inflammatory responses, thereby contributing to the pathogenesis of various inflammatory diseases (Cao et al. 2019). Notably, the antibacterial properties of *P. harmala* have been demonstrated to be effective against a broad spectrum of bacteria, including both Gram‐positive and Gram‐negative strains (Zhu, Zhao, and Wang 2022).

Under physiological conditions, the autophagy‐related protein LC3 is present in its cytosolic form, LC3I. Upon autophagy activation, LC3I undergoes lipidation to form LC3II, a process indicative of autophagosome formation. The amount of LC3II correlates with the number of autophagosomes, serving as a marker for autophagy induction. However, it is critical to understand that LC3II levels alone do not provide a complete picture of autophagic flux. Autophagic flux, a comprehensive measure of autophagic degradation activity rather than merely autophagosome accumulation, requires careful assessment. To accurately evaluate autophagic flux, one must measure not only LC3II but also p62 levels. p62, serving as an autophagy receptor, plays a pivotal role in targeting ubiquitinated proteins for degradation within lysosomes. The degradation of p62 is thus a reliable indicator of autophagic flux, offering insights into the dynamic process of autophagy beyond mere autophagosome formation. This dual assessment of LC3II and p62 expression levels is essential for a nuanced understanding of autophagic activity within cells.

**FIGURE 3:**
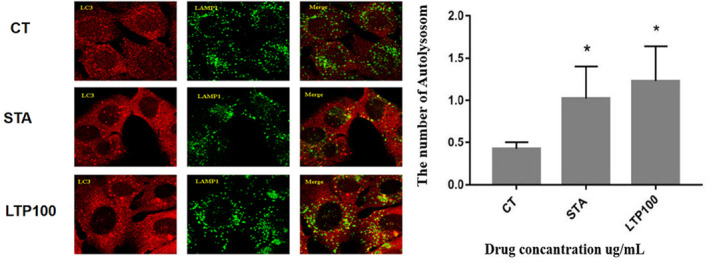
LTP induces autophagy and did not block the autophagic flux. The NRK cells that stably express GFP‐LC3 protein were treated with or without LTP 100 μg/mL for 12 h and the GFP‐LC3 fluorescence and LAMP1 were observed using confocal microscope.

**FIGURE 4:**
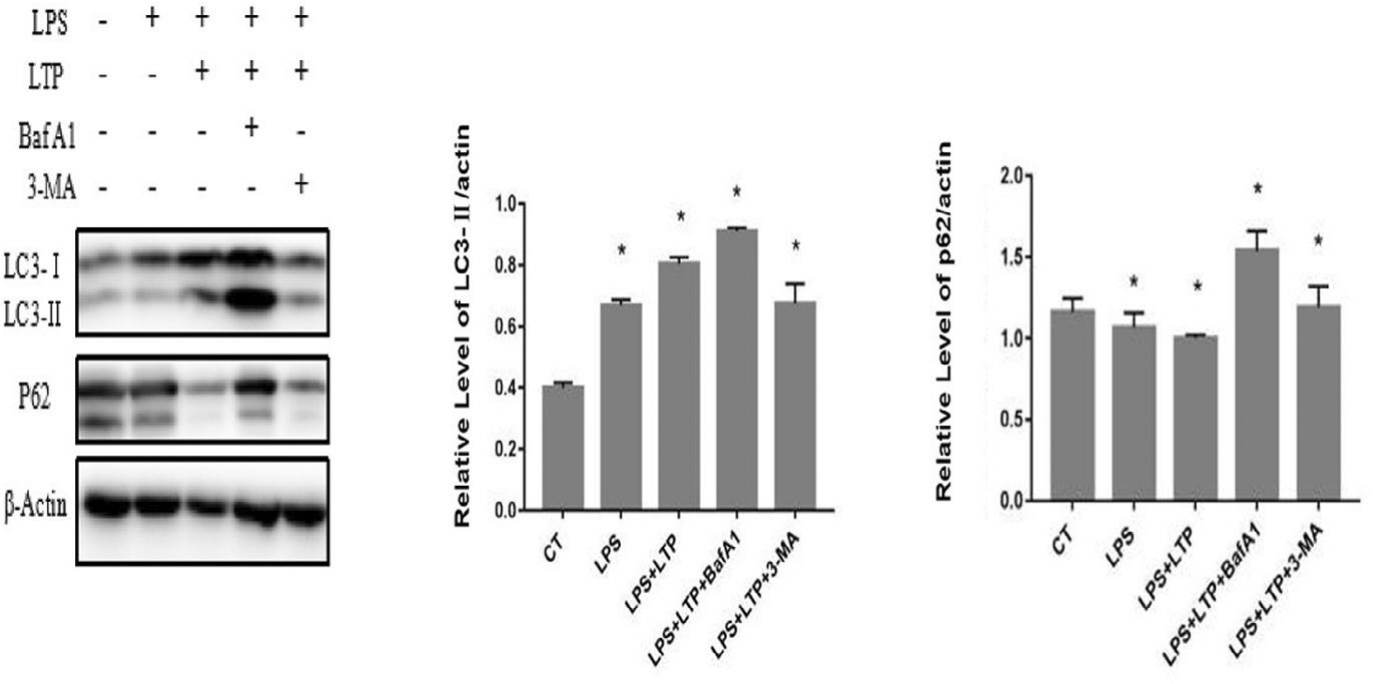
The RAW264.7 cells were treated with or without 100 μg/mL or with 50 nM Bafilomycin A1 in the absence or presence of 100 μg/mL LTP for 12 h and the relative expression of LC3‐II was determined by integrated optical density analysis.

**FIGURE 5:**
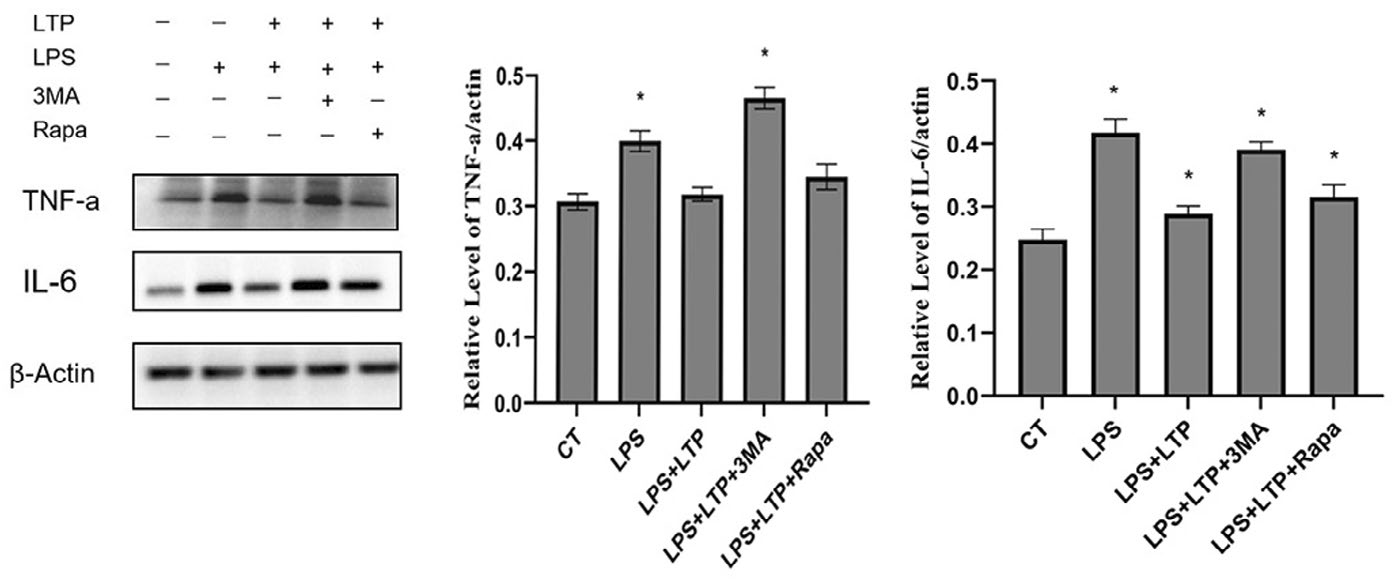
LTP suppresses the inflammatory response by inducing autophagy in macrophages. The cells underwent treatment with either 100 μg/mL or rapamycin and 3MA, with or without the presence of 100 μg/mL LTP, for a duration of 12 h. The integrated optical density analysis was utilized to determine the relative expression of LC3‐II.

The current study delves into the autophagic regulatory capabilities of LTP, elucidating its potential molecular mechanisms through a comprehensive series of in vitro experiments. Autophagy assays employed within this research revealed that LTP can activate autophagy via an mTOR‐dependent pathway. Our findings indicate a significant increase in autophagosome formation and elevated levels of LC3II expression, a hallmark protein marker for autophagosomes, following LTP treatment. Notably, this induction of autophagy by LTP does not impede the fusion of autophagosomes with lysosomes, as depicted in Figure 3. These observations robustly confirm LTP efficacy in autophagy induction. Moreover, our analysis demonstrated that the combined application of LTP and rapamycin significantly bolstered autophagy induction, surpassing the effects observed with either LTP or rapamycin alone. Further investigation into the phosphorylation status of p70S6 kinase (P‐p70S6K), a pivotal indicator of mTOR activity, revealed that LTP effectively inhibits the phosphorylation of p70S6K, mirroring the action of rapamycin. These results collectively underscore the potent autophagic modulation exerted by LTP, providing valuable insights into its underlying molecular mechanisms (Figures 4 and 5).

In the context of autophagy induced by metabolic (starvation) conditions, AMPK and autophagy are co‐induced synergistically, while mTOR is deactivated. This deactivation occurs simultaneously with autophagy. Concurrently, active mTOR is linked to inflammatory conditions and strong immune responses, which involve the transition of T cells from a dormant state. Emerging evidences demonstrated that autophagy contributes to eliminating inflammation. Thus, we then tested if the induced autophagy by LTP was related to the diminished inflammation induced by LPS in RAW264.7 cells. The results showed that the expression level of LC3 II protein in RAW264.7 cells treated with *P. harmala* polysaccharide was higher than the control group, and LC3II accumulated significantly after being treated with autophagy inhibitors bafA1. The expression of p62 decreased significantly after treated with *P. harmala* polysaccharides, and it is decreased significantly after administration of autophagy inhibitors bafA1 and 3‐MA in combination with *P. harmala* polysaccharides.

Autophagy, a complex molecular process, exhibits dual effects on cellular functions. For instance, the inhibition of autophagy, achieved through specific substances, leads to an increased expression of genes linked to inflammation in adipocytes. Conversely, activating autophagy reduces the expression of these inflammation‐associated genes, indicating autophagy's critical role in modulating a balanced inflammatory response. The exploration of autophagy's contributions to immunity and inflammation is an evolving field, blending fundamental research with practical applications. This ongoing study enriches our understanding of autophagy as both a protective and regulatory mechanism in cellular health and disease (Wu et al. 2021).

Our study demonstrated that LTP effectively mitigates inflammation in LPS‐stimulated macrophages by promoting autophagy. Specifically, we observed a significant reduction in the levels of pro‐inflammatory cytokines TNF‐α and IL‐6 in RAW 264.7 cells treated with LTP. Furthermore, when these cells were co‐treated with LTP and 3‐methyladenine (3‐MA), a known autophagy inhibitor, an increase in TNF‐α and IL‐6 levels was noted, indicating the reversal of LTP anti‐inflammatory effects. Conversely, co‐treatment with LTP and rapamycin (Rapa), an autophagy inducer, led to a decrease in TNF‐α and IL‐6 levels. These findings collectively reinforce our conclusion that LTP attenuates inflammation in RAW 264.7 macrophages, primarily through the induction of autophagy.

## Author Contributions

**Manzeremu Rejiepu:** conceptualization (equal), data curation (equal), formal analysis (equal), investigation (equal), methodology (equal), software (equal), visualization (equal), writing – original draft (equal). **Na Mi:** conceptualization (equal), data curation (equal), investigation (equal), methodology (equal), resources (equal), supervision (equal), validation (equal), visualization (equal), writing – original draft (equal), writing – review and editing (equal). **Ling Zhang:** conceptualization (equal), data curation (equal), investigation (equal), project administration (equal), resources (equal), supervision (equal), validation (equal), visualization (equal), writing – original draft (equal), writing – review and editing (equal). **Jun Shen:** conceptualization (equal), data curation (equal), formal analysis (equal), investigation (equal), methodology (equal), software (equal), visualization (equal), writing – original draft (equal). **Junqing Liang:** conceptualization (equal), data curation (equal), investigation (equal), methodology (equal), software (equal), visualization (equal), writing – original draft (equal). **Alaili Maitikabili:** conceptualization (equal), data curation (equal), investigation (equal), methodology (equal), software (equal), visualization (equal), writing – original draft (equal). **Jian Yang:** conceptualization (equal), data curation (equal), investigation (equal), methodology (equal), project administration (equal), software (equal), supervision (equal), visualization (equal), writing – review and editing (equal).

## Conflicts of Interest

The authors declare no conflicts of interest.

## Data Availability

The data that support the findings of this study are available from the corresponding author upon reasonable request.
